# Pericyte-Like Progenitors Show High Immaturity and Engraftment Potential as Compared with Mesenchymal Stem Cells

**DOI:** 10.1371/journal.pone.0048648

**Published:** 2012-11-07

**Authors:** Amina Bouacida, Philippe Rosset, Valérie Trichet, Fabien Guilloton, Nicolas Espagnolle, Thomas Cordonier, Dominique Heymann, Pierre Layrolle, Luc Sensébé, Frédéric Deschaseaux

**Affiliations:** 1 Stromalab Unité Mixte de Recherche (UMR) Université Paul Sabatier/Centre National de la Recherche Scientifique (CNRS) 5273, U1031 Institut national de la santé et de la recherche médicale (Inserm), Etablissement Français du Sang-Pyrénées-Méditerranée, Toulouse, France; 2 EA3855, Université François Rabelais, Tours, France; 3 Inserm UMR957, Physiopathologie de la Résorption Osseuse et Thérapie des Tumeurs Osseuses Primitives, Université de Nantes, Nantes, France; 4 Centre Hospitalier Universitaire Trousseau, Tours, France; University of Torino, Italy

## Abstract

Mesenchymal stem cells (MSCs) and pericyte progenitors (PPs) are both perivascular cells with similar multipotential properties regardless of tissue of origin. We compared the phenotype and function of the 2 cell types derived from the same bone-marrow samples but expanded in their respective media – pericyte conditions (endothelial cell growth medium 2 [EGM-2]) for PPs and standard medium (mesenchymal stem cell medium [MSM]) for MSCs. After 3 weeks of culture, whatever the expansion medium, all cells showed similar characteristics (MSC markers and adipo-osteo-chondroblastic differentiation potential), although neuronal potential was greater in EGM-2– than MSM-cultured cells. As compared with MSM-cultured MSCs, EGM-2–cultured PPs showed higher expression of the pericyte-specific antigen 3G5 than α-smooth muscle actin. In addition, EGM-2–cultured PPs showed an immature phenotype, with upregulation of stemness OCT4 and SOX2 proteins and downregulation of markers of osteoblastic, chondroblastic, adipocytic and vascular smooth muscle lineages. Despite having less effective *in vitro* immunosuppression capacities than standard MSCs, EGM-2–cultured PPs had higher engraftment potentials when combined with biomaterials heterotopically-transplanted in Nude mice. Furthermore, these engrafted cells generated more collagen matrix and were preferentially perivascular or lined trabeculae as compared with MSM-cultured MSCs. In conclusion, EGM-2–cultured PPs are highly immature cells with increased plasticity and engraftment potential.

## Introduction

Mesenchymal stem cells (MSCs) (also called multipotent mesenchymal stromal cells) are tissue-resident multipotential cells that give rise to bone cells, adipocytes, smooth muscle (SM) cells and hematopoietic-supportive stromal cells [1], [2], [3], [4]. They have been identified in several tissues, including bone marrow (BM), dental pulp, adipose tissue and umbilical cord blood. Such cells are selected by their capacity to adhere to plastic culture flasks and expand through fibroblastic colonies (colony formation unit fibroblasts [CFU-fs]) within media containing at least 10% fetal bovine serum (FBS) [5]. However, this *ex vivo* expansion protocol hides their true nature, which explains the growing number of works seeking to describe them directly *in vivo* in their native forms.

**Table 1:** Primers used for studies.

Gene names	5'-Forward-3' sequences	5'-Reverse-3' sequences
**ADIPOQ**	TGCCCCAGCAAGTGTAACC	TCAGAAACAGGCACACAACTCA
**ALPL**	CCTGGAGCTTCAGAAGCTCA	ACTGTG GAGACACCCATCCC
**β3-Tubulin**	ATCCGGACCGCATCATGAAC-	ACCATGTTGACGGCCAGCTT
**CNN3**	GGCTCTAGCAGGTCTGGCTAA	AATGTCAATGGTTGTATGGAATCCT
**COL2a1**	CTGCAAAATAAAATCTCGGTGTTCT	GGGCATTTGACTCACACCAGT
**DLX2**	TTCGGATAGTGAACGGGAAG	GAAGCACAAGGTGGAGAAGC
**DLX5**	GCCACCAACCAGCCAGAGA	GCGAGGTACTGAGTCTTCTGAAACC
**ELN**	AACCAGCCTTGCCCGC	CCCCAAGCTGCCTGGTG
**EMX2**	TCCAAGGGAACGACACTAGC	TCTTCTCAAAGGCGTGTTCC
**GRIA3**	ACACCATCAGCATAGGTGGACTT	ACGGCAAAGCGGAAAGC
**LEP**	CGGAGAGTACAGTGAGCCAAGA	CGGAATCTCGCTCTGTCATCA
**MAP2**	ACCGGGAGAGGCAGAATTTCCAC	AGCGCTTTTCTGGGCTCTTGGT
**MEST**	TCGAGGTCTCACCCCAGTCTT	CCACATGTCCCACAGCTCACT
**MYOC**	GGACTGCTCTGGCAACCCAGTGC	CATCTGCTGACTCCGGGTCATTTGC
**NANOG**	TGGACACTGGCTGAATCCTTC	CGTTGATTAGGCTCCAACCAT
**OCT-4 A**	AGTGAGAGGCAACCTGGAGA	GTGAAGTGAGGGCTCCCAT
**OPG**	TTCCGGAAACAGTGAATCAA	CGCTGTTTTCACAGAGGTC
**OSC**	GAGGGCAGCGAGGTAGTGAAGA	CGATGTGGTCAGCCAACTCG
**PPARγ2**	TCAGGGCTGCCAGTTTCG	GCTTTTGGCATACTC TGTGATCTC
**PTHR1**	ACATCTGCGTCCACATCAGGG	CCGTTCACGAGTCTCATTGGTG
**RANKL**	TCCCATCTGGTTCCCATAAA	GGTGCTTCCTCCTTTCATCA
**RUNX-2**	GGCCCACAAATCTCAGATCGTT	CACTGGCGCTGCAACAAGAC
**SOX2**	CCATCCACACTCACGCAAAA	TATACAAGGTCCATTCCCCCG
**SOX6**	CACCAGATATCGACAGAGTGGTCTT	CAGGGTTAAAGGCAAAGGGATA
**SOX9**	CAAGACGCTGGGCAAGCTCT	TCTTCACCGACTTCCTCCG

Native BM-MSCs express surface markers such as STRO-1, CD49a, CD73, CD146 and CD271, which led researchers to observe them mainly at the abluminal position of vessels [5], [6], [7], [8], [9]. Indeed, non-hematopoietic CD146+ cells, capable of self-renewal and supporting hematopoiesis, were identified as perivascular cells [8]. However, pericytes are also defined as populations of cells, including all perivascular cells, with similar morphologic capacity (multi-branched cells) to directly interact with endothelial cells and have common markers and properties of vascular smooth muscle cells (VSMCs; induction of contractive acto-myosine proteins) [6]. Pericytes are also multipotent cells capable of generating adipocytes, osteoblasts and chondrocytes [7], [8]. They can be characterized by nestin, aminopeptidase N (CD13), 3G5 antigen and NG2, all also described in BM-MSCs [9], [10], [11], [12], [13]. Hence, whatever their origin, BM or adipose tissue, MSCs were recently classified as pericytic cells, even if all pericytes are not MSCs [11]. Because of the close similarities with pericytes, MSCs could be at the forefront of organogenesis and tissue regeneration. For instance, recent data in mice showed that cells located at the pericytic position and at the front of invading vessels could form bone during endochondral mechanisms of skeletogenesis or after injury [14]. However, no studies have compared pericytes and MSCs from the same BM samples.

Here, we investigated the similarities between and MSC behavior and properties in MSCs and pericytic cells cultured in standard MSC medium (MSM) or endothelial cell growth medium 2 (EGM-2) for pericyte expansion, respectively. Cultured BM-derived pericytic cells showed progenitor/stem cell properties, with higher plasticity and immaturity state than standard MSCs.

**Figure 1:**
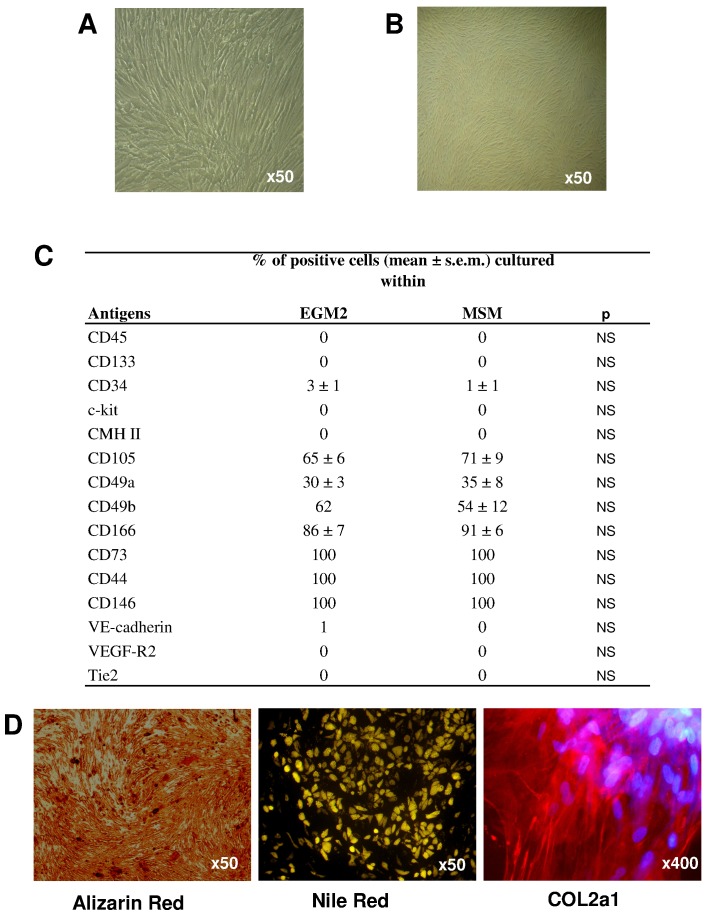
Mesenchymal stem cell (MSC) characteristics. MSC phenotype of cells cultured with endothelial cell growth medium 2 (EGM-2) and standard medium for MSCs (MSM). (**A**, **B**) Photonic microscopy of morphology of cultured cells, and (**C**) quantification by flow cytometry of expression of membrane molecules characterizing bone-marrow MSCs with antibodies against CD markers conjugated with FITC or phycoerythrine. (**D**) Alizarin-red, Nile-red and COL2a1 staining for osteoblastic, chondroblastic and adipocytic potential, respectively.

## Materials and Methods

### Cells

We collected adult human BM samples from healthy volunteers undergoing orthopedic surgery. The study followed the ethical guidelines of the University Hospital of Tours (Tours, France) and was approved by the ethics committee Comité de protection des personnes (Tours - Région Centre [Ouest-1]). Patients gave their written informed consent for use of samples. For each donor, BM mononuclear cells were plated in 1) MSM, consisting of minimum essential medium supplemented with 10% FBS (Stem-Cell Technologies, Vancouver, BC, Canada) and 1% penicillin/streptomycin (Invitrogen, Paisley, UK); or 2) EGM-2 (PromoCell GmbH, Heidelberg, Germany). On day 10, cells at 80% confluence were split and expanded (passage 1). We used cells at passage 1 or 2 from at least 3 different donors for all studies. Peripheral blood mononuclear cells (PBMCs) were obtained from informed consent donors following ethical guidelines of Etablissement Français du Sang Pyrénées-Méditerranée (Toulouse, France).

### MSC Multipotential Assay

MSC multipotentiality was determined after cell differentiation into osteoblasts, chondroblasts, or adipocytic cells as previously described [15], [16]. Osteoblastic and chondroblastic differentiation was revealed by staining with Alizarin red (Sigma-Aldrich, St. Louis, MO, USA) and anti-COL2a1 (clone 6B3, Millipore, Billerica, MA), respectively, and adipocytes by staining with Nile-red oil solution (Sigma-Aldrich). Undifferentiated cells cultured in expansion medium were a negative control. Revealing neuronal potential was as previously described [17], [18]. EGM-2– or MSM-cultured cells were cultured in neuronal induction medium, DMEM F12 with L-glutamine, 1% insulin-transferrine-selenium, and 1% penicillin–streptomycin (Invitrogen). We added Forskolin (40 µg/mL), nerve growth factor (50 ng/mL), NT3 (70 ng/mL), NT4 (100 ng/mL), and fibroblast growth factor 2 (125 ng/µL) (Euromedex, Souffelweyersheim, France). The culture medium was replaced every 3 to 4 days. Negative control cells were maintained without any induction medium. After neuronal differentiation, neurosphere-like colonies were obtained from EGM-2 conditions only. These spheres were dissociated into single cells by use of trypsin and resuspended in differentiation media (for osteogenesis, adipogenesis and neurogenesis). For all induction cultures, cells were maintained for 9 or 13 days before harvesting for RNA extraction or fixation with 4% paraformaldehyde for immunofluorescence.

**Figure 2:**
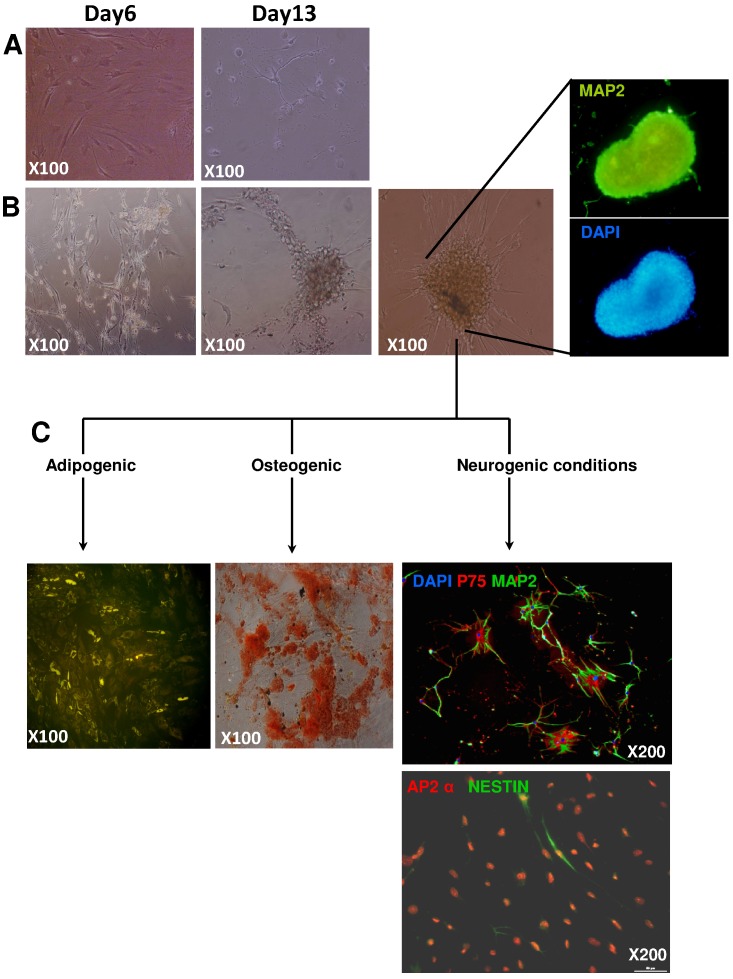
Neuronal potential of cells. Photonic microscopy of cells cultured under MSM (**A**) or EGM-2 conditions (**B**) with neuron-inducing medium on days 6 and 13, and fluorescence microscopy of human microtubule-associated protein 2 (hMAP2). After 2 weeks of neurogenic culture, only MSM culture greatly decreased MSC cell number (**A**), whereas EGM-2–derived cells still survived and aggregated, forming floating neurosphere-like structures expressing MAP2 protein (**B**). (**C**) Neurosphere-like structures underwent induction of neuronal, adipocytic and osteoblastic differentiation by culture in appropriate media after their dissociation. Sphere-derived cells re-adhered and Nile-red and Alizarin-red staining showed adipocytes and osteoblastic cells, respectively. Neurogenic culture induced the expression of neuron-specific markers p75, MAP2, AP2α and Nestin. Nuclei were stained with DAPI.

### Quantification of Bipotential CFU-fs

Sorted cell subsets were seeded at 4 to 6 cell concentrations into 96-well plates (12–24 wells/dilution). At day 10, using an inverted phase microscope colonies of more than 50 cells (CFU-fs) were counted. Then, CFU-fs from wells were induced to differentiate into adipocytes or osteoblasts as described. The frequency of responding cells at any one time was calculated from the proportion of negative wells by use of the Poisson formula.

### Assessment of Functional Potential of Pericytic Cells

Selected cells were cultured with Matrigel (Becton Dickinson, Franklin Lakes, NJ, USA) overnight before examination under a photonic microscope. Cultured cells from both conditions were co-cultured with human umbilical vein endothelial cells (HUVECs; Promocell) for 5 days, then stained with anti-von Willebrand Factor (vWF) (Dako, Trappes, France) and anti-αSM actin antibodies.

**Figure 3:**
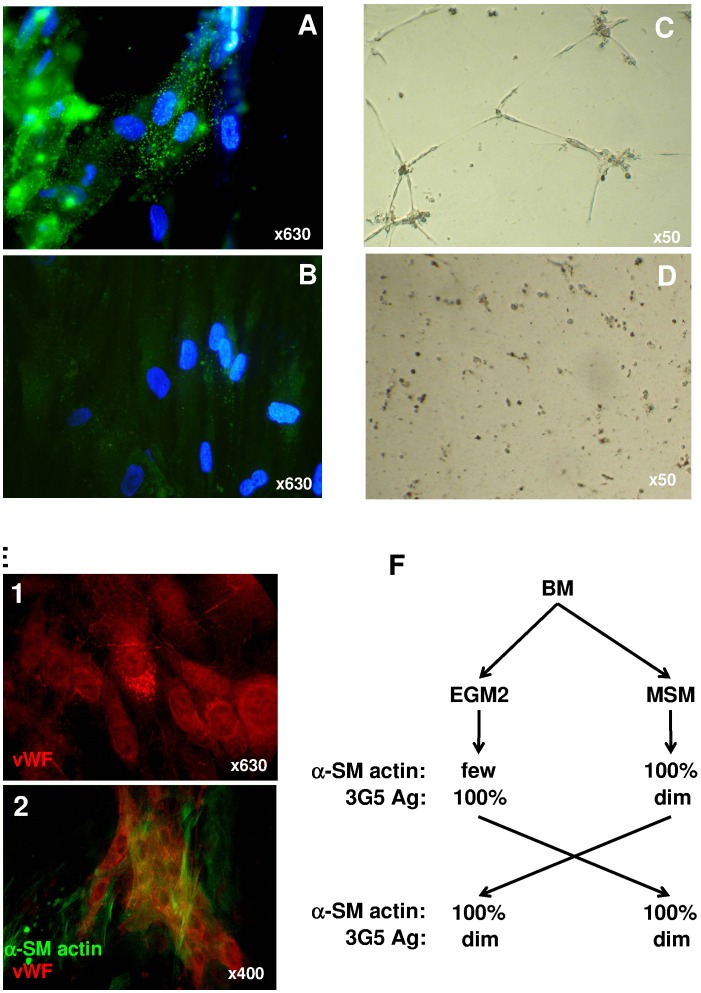
Pericyte phenotype and function. Assessment of pericyte phenotype by pericyte-specific 3G5 antibody (**A**, **B**) and function by seeding onto matrigel (**C**, **D**, **E**) in cells cultured with EGM-2 (**A, C**) or MSM (**B, D**). Cultured human umbilical vein endothelial cells (HUVECs) were incubated with anti-von Willebrand factor (vWF) antibody revealing Weibel-Palade bodies (**E1**). EGM-2–cultured cells were seeded onto HUVECs and incubated for 5 days and labeled with (**E2**) anti-α-smooth muscle (SM)-actin and anti-vWF factor antibodies. (**F**) Proportion of EGM-2– or MSM-cultured cells (from the same bone-marrow [BM] sample) expressing αSM actin and 3G5 antigen (3G5 Ag) before or after priming into the opposite medium (MSM or EGM-2, respectively).

### Flow Cytometry Studies

Both cell types were examined by flow cytometry. Cells were washed in phosphate buffered saline/human serum albumin solution, and the following primary antibodies conjugated to phytoerythrin (PE) or fluorescein isothiocyanate (FITC) were added at the appropriate concentrations: stem cell factor receptor (CD117), CMH-II, and CD105 (Diaclone, Besançon, France); mouse monoclonal antibody (Mab) anti-human CD34, CD45, and CD146 (Millipore); Mabs anti-human CD133 (Miltenyi Biotec), anti-human vascular endothelial growth factor-type 2 receptor (anti-VEGF-R2), CD49a, CD49b, CD166, CD73, CD44, and Tie2 (Becton Dickinson); and rabbit polyclonal anti-human CD144 (VE-cadherin) (Valbiotech, Paris, France). Antibodies of the same isotype were used as negative controls (Diaclone, Immunotech, Becton Dickinson and Millipore). Cells were passed through the Becton Dickinson FACSort (Mountain View, CA) equipped with the Cellquest software program. The fluorescence histogram for each monoclonal antibody was displayed with that of the corresponding control antibody.

**Figure 4:**
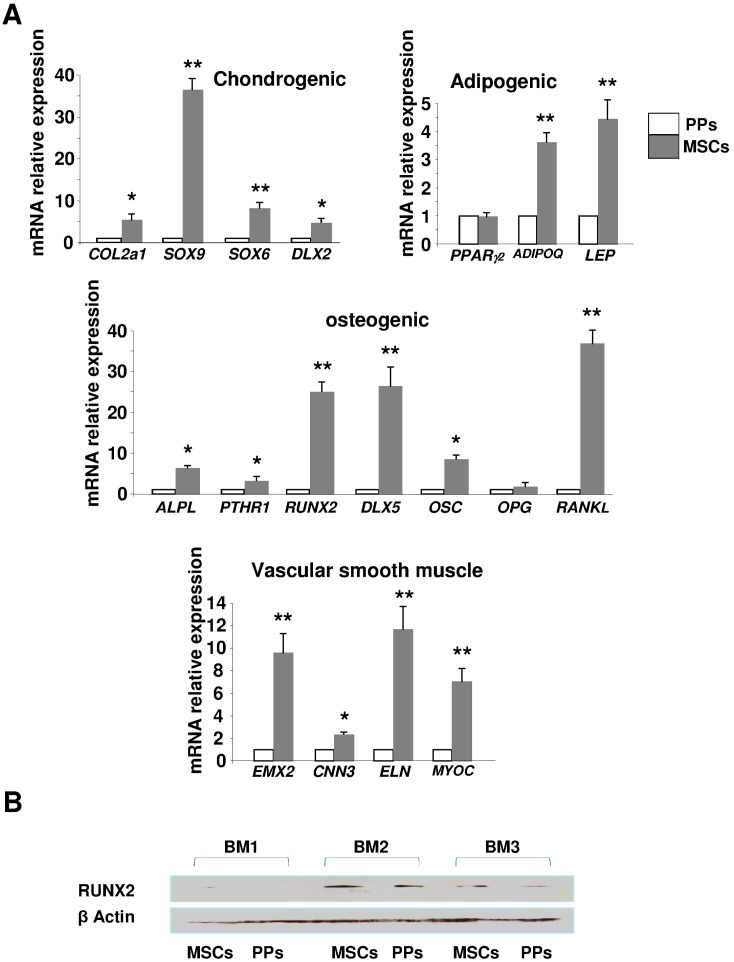
Expression of mesenchymal lineage markers. (**A**) Quantitative RT-PCR of relative mRNA expression of markers of chondrogenesis (*COL2a1*, *SOX9*, *SOX6*, *DLX2*), adipogenesis (*PPARγ2*, *ADIPOQ*, *LEP*), osteogenesis (*ALPL*, *PTHR1*, *RUNX2*, *DLX5*, *OSC*, *OPG*, *RANKL*), and vascular smooth muscle (VSM) (*EMX2*, *CNN3*, *ELN*, *MYOC*). Expression was relative to that of *GAPDH* and depicted as fold change relative to that for pericyte progenitors (PPs). Data are mean±SEM from 6 experiments. *p<0.01; **p<0.001 compared with PPs. (**B**) Western blot analysis of protein level of RUNX2 in 3 different BM productions (BM1, BM2, and BM3) of bone-marrow MSCs and PPs. β-actin was a positive internal control.

After expansion, EGM-2– or MSM-cultured cells were cultured in chamber slides, and confluent cells were stained with 3G5 antibody (produced from the ATCC cell line and purified by RD-Biotech, Besançon, France) or by anti-αSM actin (clone 1A4, Sigma-Aldrich), and then at 37°C for 45 min with FITC-labeled goat anti-mouse or donkey anti-rabbit secondary antibody (Invitrogen). After neuronal differention, cells were incubated with the antibodies anti-Nestin, -AP2α, -p75, -MAP2, -SOX10, -SLUG and anti-NeuN (see supporting information). Positive cells were counted and compared to total cell counts for percentage positive cells.

### Real-time RT-PCR

Total RNA was extracted from differentiated and undifferentiated cells and purified with use of the RNAeasy purification kit (Qiagen, Courtaboeuf, France). Reverse transcription involved the Takara Kit (St. Germain en Laye, France) in a final volume containing 1 µg RNA. The reagents in 20 µL PCR included 1 X SYBR GREEN mix (Biorad, Marnes-la-Coquette, France) and 25 ng cDNA. The primers are in Table 1. All real-time PCR reactions were performed in duplicate. mRNA expression was normalized to that of GAPDH.

**Figure 5:**
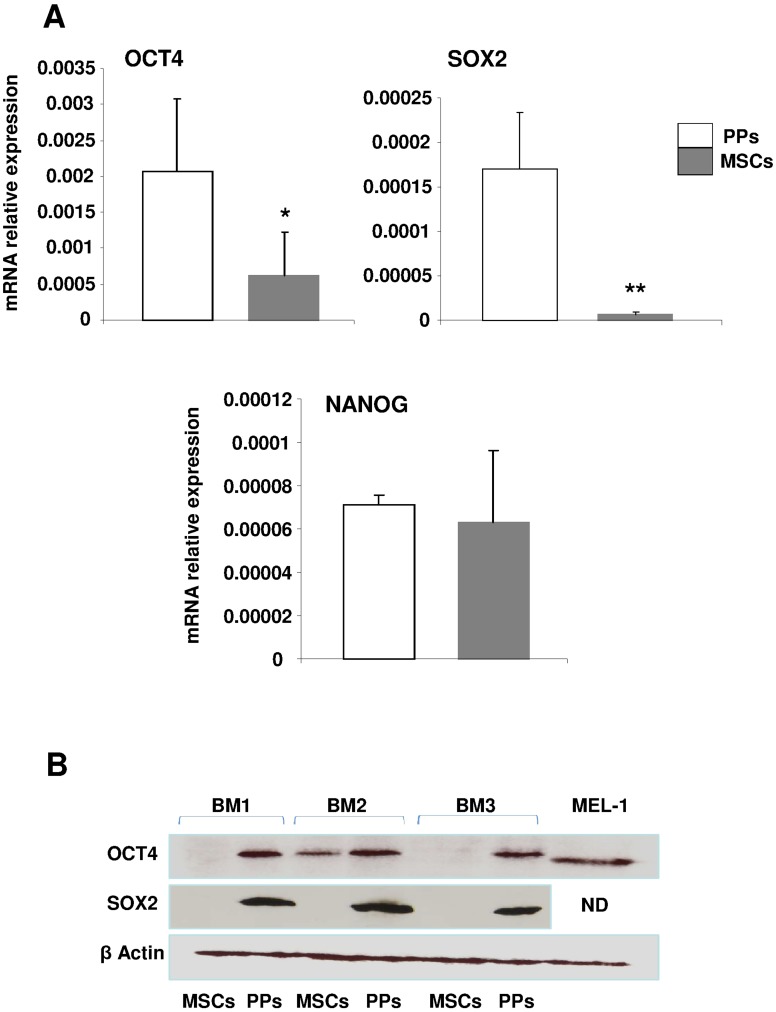
Expression of stemness markers. Quantitative RT-PCR (**A**) and western blot (**B**) analysis of the mRNA and protein expression, respectively, of stemness specific markers OCT4A, SOX2 and quantitative RT-PCR analysis of NANOG mRNA expression. Data are mean±SD from 3 experiments. *p<0.01; **p<0.001 compared with PPs. Protein extracts of cells from 3 different BM cultures (BM1, BM2, BM3) of PPs vs MSCs were tested. β-actin was a positive internal control. ND: not determined. MEL1: protein extract of human embryonic stem cell line MEL1 as positive control.

### Western Blot Analysis

Proteins were extracted, separated by 10% Tris-glycine on SDS-PAGE, and transferred to nitrocellulose membrane (Amersham Biosciences, Saclay, France), then incubated with anti-OCT4, anti-SOX2 and anti-RUNX2 antibodies (Abcam), 1 µg/mL, then peroxidase-conjugated mouse anti-rabbit IgG antibody (Sigma-Aldrich) and visualized by enhanced chemiluminescence (Thermo Fisher, Courtaboeuf, France). β-actin was an internal control (Sigma-Aldrich).

**Figure 6:**
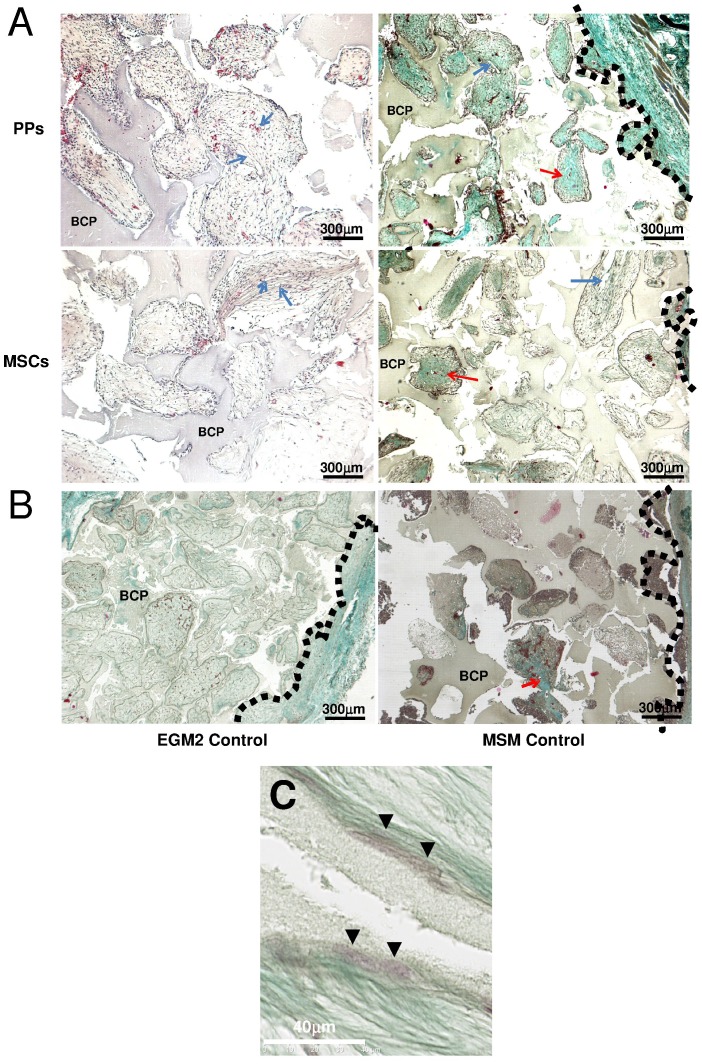
*In vivo* investigation of stem/progenitor cell seeding in mice. EGM-2–derived PPs or MSM-derived MSCs were seeded into biphasic calcium phosphate (BCP) biomaterial discs and inserted in the back of mice (**A**). Several BCP discs were not loaded with cells as negative controls (**B**). After 8 weeks, all discs were removed and evaluated for collagen matrix deposition within transplants and human cell engraftment. Vessels and bone collagen were observed after hematoxylin (left) or Masson’s trichrome (right) staining, respectively. Dotted black lines indicate the outer border of discs. Red arrows show vessels, and blue arrows show bone matrix. (**C**) BCP discs loaded with EGM-2–cultured PPs show trabeculae lined with cells engulfed within matrix on Masson’s trichrome staining (green fibers).

### Animal Study and Histology

All animal study procedures were performed in accordance with a protocol approved by the local committee for animal care and ethics (French ethics committee CEEA.PdL.06 and local veterinary services (license n°C44015)). Macroporous biphasic calcium phosphate (BCP) ceramic discs, 8 mm in diameter and 3 mm thick (Biomatlante SA, Vigneux de Bretagne, France), were composed of 20 hydroxyapatite and 80 wt.% β-tricalcium phosphate and sterilized by gamma irradiation (total porosity: 70% to 75%; macroporosity: 400 µm). All experiments involved a minimum of 5 animals per group. In total, 200 µL medium containing 7.5×10^5^ human MSCs were seeded onto BCP discs, and after their absorption, 1 mL medium was added (experiments were repeated with MSCs from 3 different donors). The cells were cultured in the tested media for 7 days in low attachment plates, and medium was refreshed twice weekly. Five samples were used for each condition.

**Figure 7:**
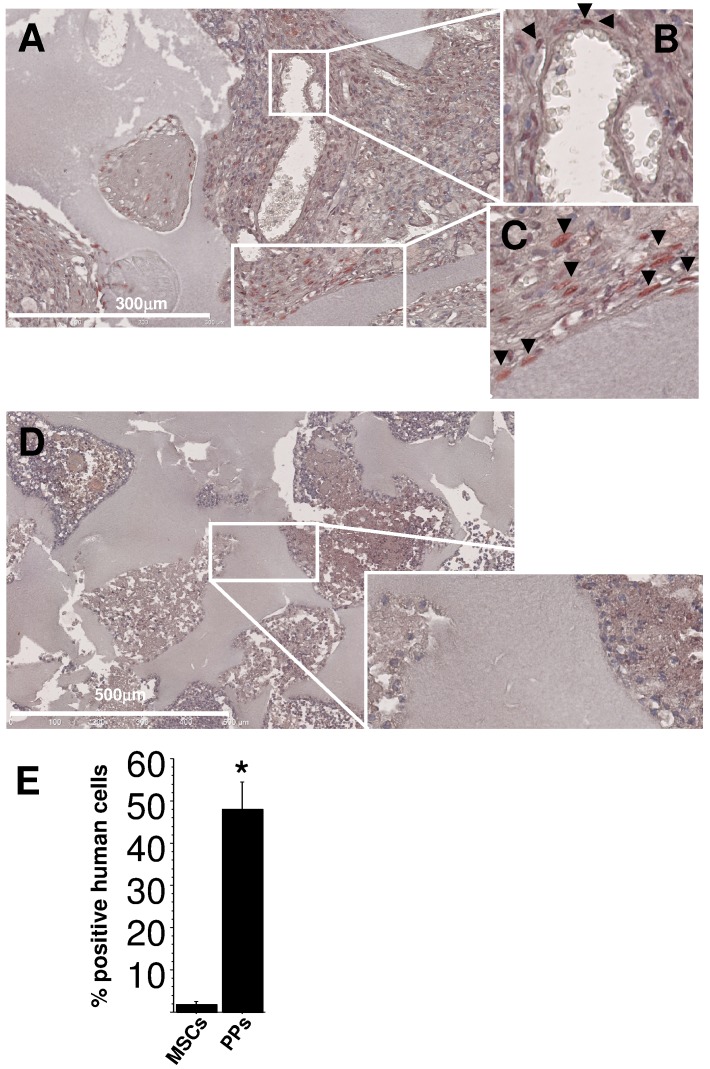
*In vivo* detection of cells of human origin. Alu sequences were checked in cells from microsections of BCP transplants seeded with PPs cultured with EGM-2 (**A–C**) or MSCs with MSM (**D**). (**A**) Human cells were depicted by red staining in nuclei (arrowheads). (**B**) and (**C**) enlargement of the squared area in (**A**). Human cells lined BCP trabeculae, formed fibrous tissue, or were at the abluminal position of vessels (**B, C**). (**D**) Very few cells of human origin were detected with seeding of MSM-cultured MSCs. (**E**) Quantification of human vs host cells involved counting the number of Alu+ nuclei (*p<0.001) in more than 10 fields per transplant and 3 transplants.

Cultured BCP discs were subcutaneously implanted in the backs of NMRI nude male mice (Elevage Roger Janvier, Le Genest Saint Isle, France). A 1-cm-long incision was made on each side of the mouse back, and blunt dissection was used to separate the skin from subcutaneous connective tissue to form pockets into which discs were inserted. The mice were killed after 8 weeks by lethal intracardiac injection of thiopental, and the implants were retrieved. The explants were processed according to standard operating procedures for non-decalcified histology. Samples were decalcified in EDTA, dehydrated and embedded into paraffin; 100-µm-thick cross sections were obtained by use of a diamond-saw microtome (Leica SP 1600, Leica Microsystems, Nanterre, France), stained with hematoxylin or Masson’s trichrome and analyzed by light microscopy.

The human origin of cells within the transplants was confirmed by *in situ* hybridization of human-specific genomic repetitive sequence as described [19]. A locked nucleic acid (LNA) probe was designed within the human Alu-Sb2 subfamily consensus sequence (Genbank no. HSU14570) with the advanced online design software of Exiqon (Vedbaek, Denmark). The LNA-Alu probe was labeled with Digoxigenin (DIG) at the 5′ and 3′ ends. Deparaffinized and rehydrated sections were treated with 1 mg/ml pepsin in 0.1 N HCl at 37°C for 30 min. After a wash with PBS, 70 nM Alu-LNA probe was added to tissue sections in 2X SSC (300 mM sodium chloride, 30 mM trisodium citrate, pH 7.4), 50% deionized formamide and 10% dextran sulfate. Denaturation of DNA was carried out at 95°C for 5 min, followed by hybridization at 37°C for 2 hr. After 2X SSC 50% deionized formamide washes, the hybridization of LNA-Alu probe was revealed with horseradish peroxydase-conjugated anti-DIG antibody conjugated (1/50e; Roche Diagnostic France) with use of an AEC staining kit (Sigma Aldrich). Nuclei were counterstained with hematoxylin and sections were dehydrated and mounted with Faramount medium (Dako). Sections of human osteosarcoma in muscle of nude mouse were positive and negative controls during *in situ* hybridization with the LNA-Alu probe.

### Statistical Analysis

Data are expressed as mean ± SEM and were analyzed by nonparametric Kruskal-Wallis test. Two-sided P<0.05 was considered statistically significant.

## Results

### EGM-2-derived Cells are Highly Plastic Progenitors

We split BM mononuclear cells from donors and cultured them in *in vitro* medium adapted for pericytes, EGM-2 [20], and compared results to culture in medium for MSCs, MSM. Culture in EGM-2 produced CFU-fs. EGM-2- and MSM-cultured cells did not differ in lag phase before colony appearance, proliferation or CFU-f number, size or number of bipotential CFU-f (adipocyte and osteoblastic potentials) (data not shown). As well, EGM-2– and MSM-cultured cells did not differ in MSC phenotypic characterization, including morphologic features, membrane molecules or ability to generate osteoblasts, chondroblasts and adipocytes (Figure 1). However, when EGM-2-derived cells were cultured under neurogenic conditions, cells aggregated to form MAP2+ neurosphere-like structures, which were never observed with standard MSCs (Figure 2). After their dissociation, the cells were assessed for adipocyte and osteo-chondroblastic multipotentiality. We observed lipid-droplet–containing cells or mineralization, although with low efficiency. In addition, after prolonged neurogenic induction, we observed neuronal cells. Of note, in contrast to standard MSCs, EGM-2–cultured MSCs showed a significant increase in commitment toward a neuronal lineage (Figure 2 & Figure S1).

### EGM-2 Culture Consistently Produced a Pericyte Phenotype

We assessed the perivascular/pericytic phenotype and function of cells in our *in vitro* conditions. We characterized pericytes with anti-αSM actin and -3G5 antibodies, 2 markers used for pericyte characterization [6]. 3G5 staining was greater for EGM-2– than MSM-cultured MSCs (Figure 3A, B). Because pericytes can themselves (and without endothelial cells) form interconnected networks between them when seeded on basement membrane proteins found in Matrigel [21], [22], we tested this potential after overnight incubation. As compared with MSM-cultured MSCs, only EGM-2–cultured cells organized into pericyte-like networks (Figure 3C, D). As well, when seeded with HUVECs, EGM-2–cultured cells were organized near HUVECs as αSM actin+ cells recovering von Willebrand factor+ endothelial cells and forming structures resembling tubes (Figure 3E). Therefore, EGM-2-cultured cells exhibited a pericyte phenotype and function that was more marked than with MSM-cultured MSCs. Surprisingly, almost all MSM-cultured but only a few EGM-2–cultured PPs were positive for αSM actin.

We wondered whether the media were capable of modulating 3G5 antigen and αSM actin expression, so we cultured cells in MSM until confluence and shifted them to EGM-2 and vice versa (Figure 3F). Whatever the combination after culture in MSM, all cells remained 3G5_dim_ but αSM actin+. Therefore, the standard MSC phenotype persisted in MSCs, whatever the culture conditions used, as compared with EGM-2 cells, which showed modified expression of 3G5 antigen or αSM actin in MSM culture and rendered them phenotypically indistinguishable from standard MSCs. Because the αSM actin molecule is a differentiation-stage–dependent marker of VSMC lineage, EGM-2-cultured PPs might be phenotypically more immature than standard MSCs [6]. Therefore, regarding these data EGM-2 derived cells were hereafter referred to as pericyte progenitors (PPs).

### EGM-2-cultured PPs are more Immature than are MSM-cultured MSCs

Classical MSCs express αSM actin and several lineage markers because of their differentiation potential [23]. In addition to αSM actin for the VSMC lineage, osteoblastic, chondroblastic and adipocytic lineages are revealed by the expression of the molecules RUNX2, SOX6 and PPARγ, respectively [23]. The mRNA expression of all genes characterizing chondroblasts (*COL2A1*, *SOX9*, *SOX6* and *DLX2*), adipocytes (*AdipoQ*, *LEP*), osteoblasts (*ALPL*, *PTHR1*, *RUNX2*, *DLX5* and *OSC*), and VSM (*EMX2*, *CNN3*, *ELN*, *MYOC*) was lower in EGM-2– than MSM-cultured cells, but the mRNA level of *PPARγ2* was comparable (Figure 4A).

RUNX2 (a master gene of skeletogenesis) is expressed in BM-MSCs [1]. Hence, it typifies BM-MSCs as skeletal stem cells (i.e., cells generating all BM mesenchymal lineages). We found RUNX2 protein level decreased in EGM-2-cultured PPs, which confirmed the RT-PCR mRNA results (Figure 4B).

We focused on the molecules NANOG, OCT4 and SOX2 that are specific to immaturity/stemness characterizing embryonic and induced pluripotent stem cells (ES and iPS, respectively) [24]. OCT4 has several isoforms, mainly OCT4A and OCT4B, and only OCT4A contributes to the stemness properties found in ES and iPS cells [25]. The mRNA expression of *OCT4A* and *SOX2* was greater (2- and 20-fold increase, respectively) in EGM-2– than MSM-cultured cells (Figure 5A), but the 2 cells types did not differ in *NANOG* expression. The protein level of OCT4A was consistently found in EGM-2 culture but was not or was barely detected in MSM-cultured MSCs; the protein expression of SOX2 was detected only in EGM-2–cultured PPs (Figure 5B). Furthermore, upregulated OCT4A and SOX2 expression was associated with decreased RUNX2 expression in EGM-2–cultured PPs, which was opposite to that in MSM-cultured cells. Therefore, with the increased protein expression of stemness markers and concomitant decrease in lineage markers, EGM-2-cultured PPs were phenotypically more immature than MSM-cultured MSCs.

### EGM-2-cultured PPs Show Strong Engraftment Potential

We investigated engraftment capacity *in vivo* in nude mice. BM cells were cultured in EGM-2 or MSM and then seeded into bone substitutes (BCP discs). After adherence, biomaterials were inserted in mice for 8 weeks. Collagen matrix inside the biomaterials was greater for discs seeded with EGM-2– than MSM-cultured cells (Figure 6). Furthermore, discs seeded with EGM-2–cultured PPs showed cells lining trabecules and engulfed within extracellular matrix as compared with discs seeded with MSM-cultured MSCs (Figure 6C). In seeking the human origin of cells with Alu sequences, we observed numerous human cells with a mesenchymal appearance only within EGM-2–derived transplants (Figure 7A). In contrast to EGM-2–cultured PPs, few MSM-cultured MSCs of human origin were detected (48.1%±6 vs. 1.8±0.7 human to host cells, respectively, p<0.001) (Figure 7D and E). We found 3 types of localizations in EGM-2–derived transplants: the perivascular position, within the fibrous matrix and along BCP trabeculae (most). Interestingly, despite their strong engraftment potential, PPs had significantly lower effective immunosuppression properties than MSCs *in vitro* (Figure S2).

## Discussion

The abluminal position seems to be the preferential niche for several types of multipotential cells such as MSCs and PPs, whatever the tissue of origin. This position allows them to maximize all of their properties (i.e., organogenesis, tissue renewal and regeneration post-injury). MSCs and PPs share numerous properties and are phenotypically similar. Here, we aimed to compare these 2 types of cells from human BM. *In vitro*-derived PPs cultured in EGM-2 showed more plasticity, stronger expression of stemness markers (immaturity) and more engraftment potential than classically cultured MSCs. Notably, at 8 weeks post-transplantation in mice, EGM-2–derived PPs showed strong capacity for collagen matrix formation and were found mainly in perivascular and trabecular niches.

Despite the strong engrafment potential of PPs in mice, PPs showed *in vitro* immunosuppression but at a lower extent than for MSCs. The reactivity of PPs with immune cells was previously not investigated and deserves further study.

Phenotypes of BM-derived PPs and MSCs are similar except in 3G5 and αSM actin expression. 3G5 is used to characterize or select pericytes [6], [13]. Despite any comparison between BM MSCs and BM PPs, 3G5 was also found expressed by MSCs, although in their native form [26], [27]. 3G5 was found in cultured MSCs, but its expression seems to be downregulated with standard culture [27]. This finding is in agreement with our results showing that standard culture conditions (MSM) irreversibly decreased 3G5 antigen expression. Furthermore, such decrease was followed by induced expression of αSM actin protein.

αSM actin is also widely used as a pericyte and MSC marker whatever the tissue of origin [28]. Our data showed few cells positive for αSM actin when expanded in EGM-2. This discrepancy can be explained by the immaturity of EGM-2 derived cells. Indeed, in VSM differentiation processes, αSM actin is an early-differentiation stage marker, which implies that more immature cells do not express it [29], [30]. This suggestion was confirmed recently by several reports showing that pericyte precursors from iPS or perivascular progenitor cells do not express αSM actin [31], [32]. However, the αSM actin gene promoter contains the CArG box, a cis-element regulating its expression, as well as those of the VSMC lineage (e.g., SM22α and CD49a) [33]. The CarG element specifically binds the serum response factor, which is stimulated by serum [34]. Because serum is in much higher content in standard medium for MSC expansion (10% FBS) than in EGM-2 (2% FBS), standard culture conditions are thus more likely to induce αSM actin expression than EGM-2 medium. Nevertheless, despite the absence of αSM actin expression, EGM-2-derived PPs could give rise to cells with pericyte capacities because they can cover endothelial cells and form a network in Matrigel, even in the absence of endothelial cells [21], [22]. Interestingly, these pericytes became αSM actin+ when interacting closely with HUVECs in co-cultures, which suggests their induction from endothelial cells as previously described [35]. Therefore, despite the reduced αSM actin expression (mechanisms that remain to be clarified), EGM-2–cultured PPs showed pericyte properties.

The reduced αSM actin expression in PPs suggested that they were highly immature. This hypothesis was confirmed by the low expression of a panel of lineage markers concomitant with the upregulated expression of stemness markers. Indeed, OCT4 and SOX2 proteins were consistently expressed with EGM-2 culture, and their expression was opposite to that of the osteoprogenitor marker RUNX2. This finding agrees with OCT4 and SOX2 being stemness proteins allowing self-renewal of ES cells, with greatly decreased expression when cells commit to a lineage [36]. The finding is also in line with a recent report demonstrating that early-passage MSCs, or MSCs with p21 knocked out, showed upregulated expression of OCT4, SOX2 and NANOG to the detriment of tissue-specific markers and that knock-down of OCT4 or NANOG enhanced the expression of the tissue markers [37]. The authors used 16.6% FBS and hypoxic (1% O_2_) conditions for expansion of MSCs. Therefore, the culture conditions are crucial for the stem-cell potential of MSCs. Here, we showed that pericyte-specific culture conditions generated PPs with more immature features that standard MSCs.

Despite the well-known plasticity of standard MSCs, the immature state of EGM-2–cultured PPs was associated with greater differentiation potential because they yielded reliable neuronal differentiation, whereas most MSM-cultured MSCs did not survive after induction. Interestingly, after induction, EGM-2-expanded PPs formed aggregates resembling neurospheres consisting of cells capable of generating osteoblasts, adipocytes and neuronal cells. Cells forming neurospheres can be stem-cell–like cells [38]. This phenomenon was previously reported for PPs from post-natal rat aorta and perivascular progenitor cells from human adult vena saphena, which further demonstrates a similar phenotype to the PPs we describe [32], [39].

Therefore, as compared with standard culture conditions for MSCs, culture with EGM-2 selects PPs with broader potentiality, probably because of their immature state. However, we cannot exclude that BM contains several types of non-hematopoietic multipotential cells and *in vitro* conditions could select one type for their expansion. Nor can we exclude that highly immature non-hematopoietic multipotential cells can be expanded better under EGM-2 than standard culture conditions or, in other words, standard conditions may allow for more restrictive stem-cell potentials, probably because of proteins in serum. Indeed, we showed the irreversible gain in αSM-actin expression when cells were cultured under standard conditions (Figure 3F).

In conclusion, EGM-2 can be used for expansion of highly immature PPs with strong differentiation and engraftment capacities. These conditions can also allow for studying BM adult stem-cell biology and may improve *in vitro* protocols for clinical use. However, several questions remaining to be solved include the origin of EGM-2-derived PPs, their true stemness states, their complete differentiation capacities and the molecular mechanisms yielding such properties.

## Supporting Information

Figure S1 **Neuron differentiation potential of cells cultured in endothelial growth medium 2 (EGM-2) and mesenchymal stem cell medium (MSM).** (**A**) Quantitative RT-PCR analysis of mRNA level of neuronal markers *MAP2*, *β3Tubulin* or *β3T*, *MEST*, *GRIA3* and (**B**) *in situ* immunofluorescence (**Table**) of SLUG, NeuN, MAP2, and SOX10 levels. Differentiation potentials are at the bottom of the panel. For neuronal differentiation characterization, cells were fixed and permeabilized after neuronal induction, then incubated at 37°C for 1 h with the primary antibody mouse anti-human Nestin (hNestin) clone 196908, anti-SLUG or anti-SOX10 (R&D Systems, Lille, France); mouse anti-hNeuN (Sigma-Aldrich); mouse anti-human microtubule-associated protein 2 (hMAP2) clone M-9942 (Sigma-Aldrich); rabbit anti-hP75 (nerve growth factor receptor [NGFR]) (ab-8878, Abcam, Paris); rabbit anti-hAP2α (Abcam, ab108311) and then at 37°C for 45 min with FITC-labeled goat anti-mouse or donkey anti-rabbit secondary antibody (Invitrogen). Positive cells were counted and compared to total cell counts for percentage positive cells.
(TIF)

Figure S2 **Immunosuppression potential assay of PPs and MSCs.** For in vitro immunosuppression assay 50×10^3^ cells cultured in EGM-2 or MSM were co-cultured with 100×10^3^ CD3+ T cells isolated from PBMCs by use of a T-cell purification kit (Miltenyi Biotec, Bergish Gladbach, Germany). We used 3 donors for MSCs or PPs and 3 donors for PBMCs. Before the co-cultures, CD3+ cells were labelled with 5-(and-6)-carboxyfluorescein diacetate, succinimidyl ester (CFSE, Invitrogen) as fluorescent cell-tracing reagent. T cells were then activated with CD3+CD28+ microbeads (Miltenyi Biotec). After 5 days, all cells were recovered, and T cells were stained with anti-CD3 and anti-CD45 antibodies (Miltenyi Biotec). The proportion of cycling CD3+CD45+ T cells was quantified by fluorescence decrease of CFSE as compared with both uncycling cells (non-stimulated cells) and stimulated T cells cultured without MSCs or PPs. Analysis involved use of the Cyan™ flow cytometer (Beckman Coulter, Villepinte, France) and Kaluza™ software. The percentage of CD3+ T lymphocytes proliferating during the co-cultures (LyT + MSCs or LyT + PPs) were calculated. Stimulated T lymphocytes without MSCs or PPs (LyT) were used as control cells, for 100% of cycling cells. Data are mean ± SEM from 3 experiments.
(TIF)
