# Supplementary figures and images for: Pericyte-Like Progenitors Show High Immaturity and Engraftment Potential as Compared with Mesenchymal Stem Cells
Source: PLoS One. 2012 Nov 7;7(11):e48648. doi: 10.1371/journal.pone.0048648 (PMC3492496; doi:10.1371/journal.pone.0048648)

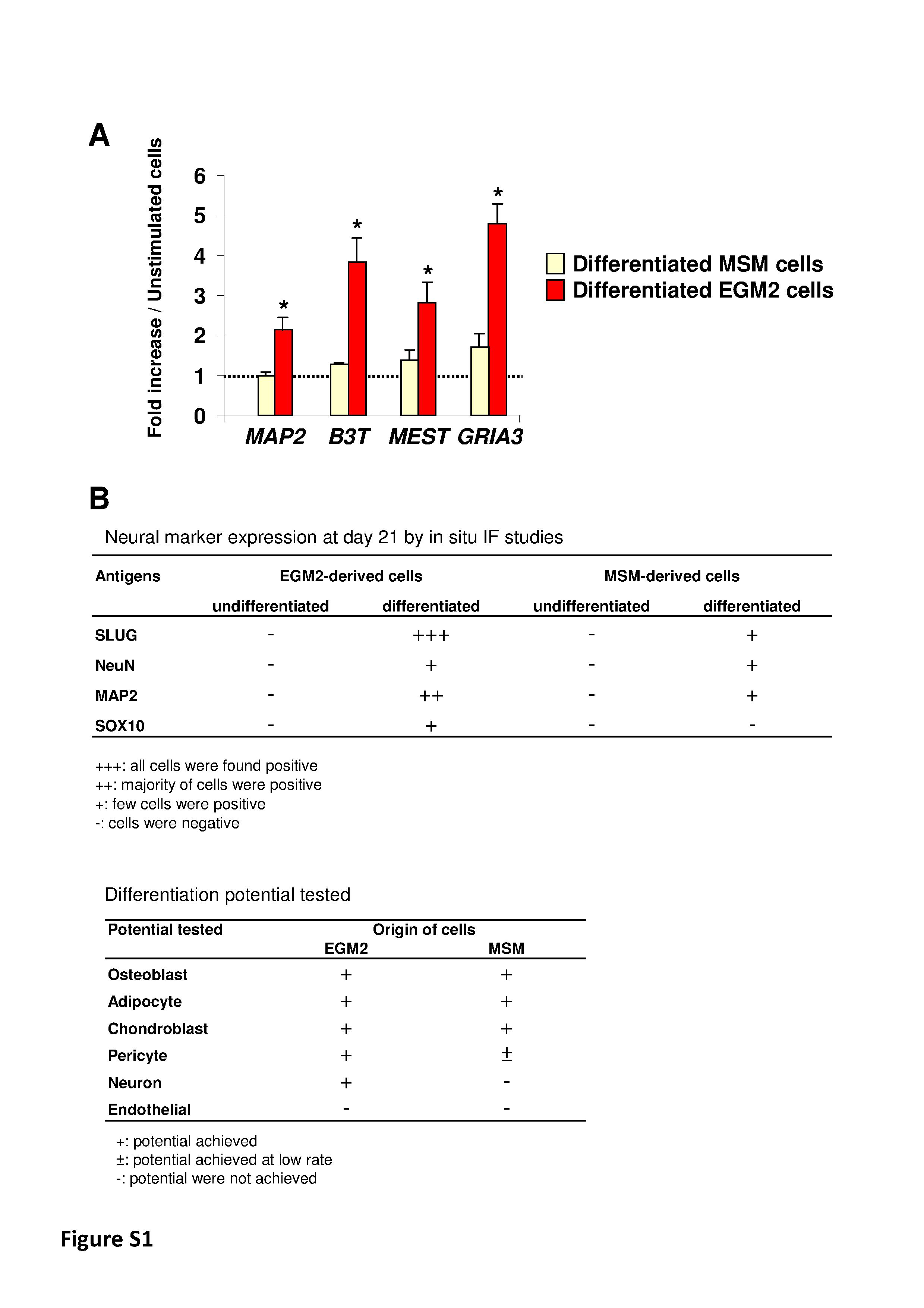

Supplement: Figure S1 — Neuron differentiation potential of cells cultured in endothelial growth medium 2 (EGM-2) and mesenchymal stem cell medium (MSM). (A) Quantitative RT-PCR analysis of mRNA level of neuronal markers MAP2, β3Tubulin or β3T, MEST, GRIA3 and (B) in situ immunofluorescence (Table) of SLUG, NeuN, MAP2, and SOX10 levels. Differentiation potentials are at the bottom of the panel. For neuronal differentiation characterization, cells were fixed and permeabilized after neuronal induction, then incubated at 37°C for 1 h with the primary antibody mouse anti-human Nestin (hNestin) clone 196908, anti-SLUG or anti-SOX10 (R&D Systems, Lille, France); mouse anti-hNeuN (Sigma-Aldrich); mouse anti-human microtubule-associated protein 2 (hMAP2) clone M-9942 (Sigma-Aldrich); rabbit anti-hP75 (nerve growth factor receptor [NGFR]) (ab-8878, Abcam, Paris); rabbit anti-hAP2α (Abcam, ab108311) and then at 37°C for 45 min with FITC-labeled goat anti-mouse or donkey anti-rabbit secondary antibody (Invitrogen). Positive cells were counted and compared to total cell counts for percentage positive cells. (TIF) [file pone.0048648.s001.tif]

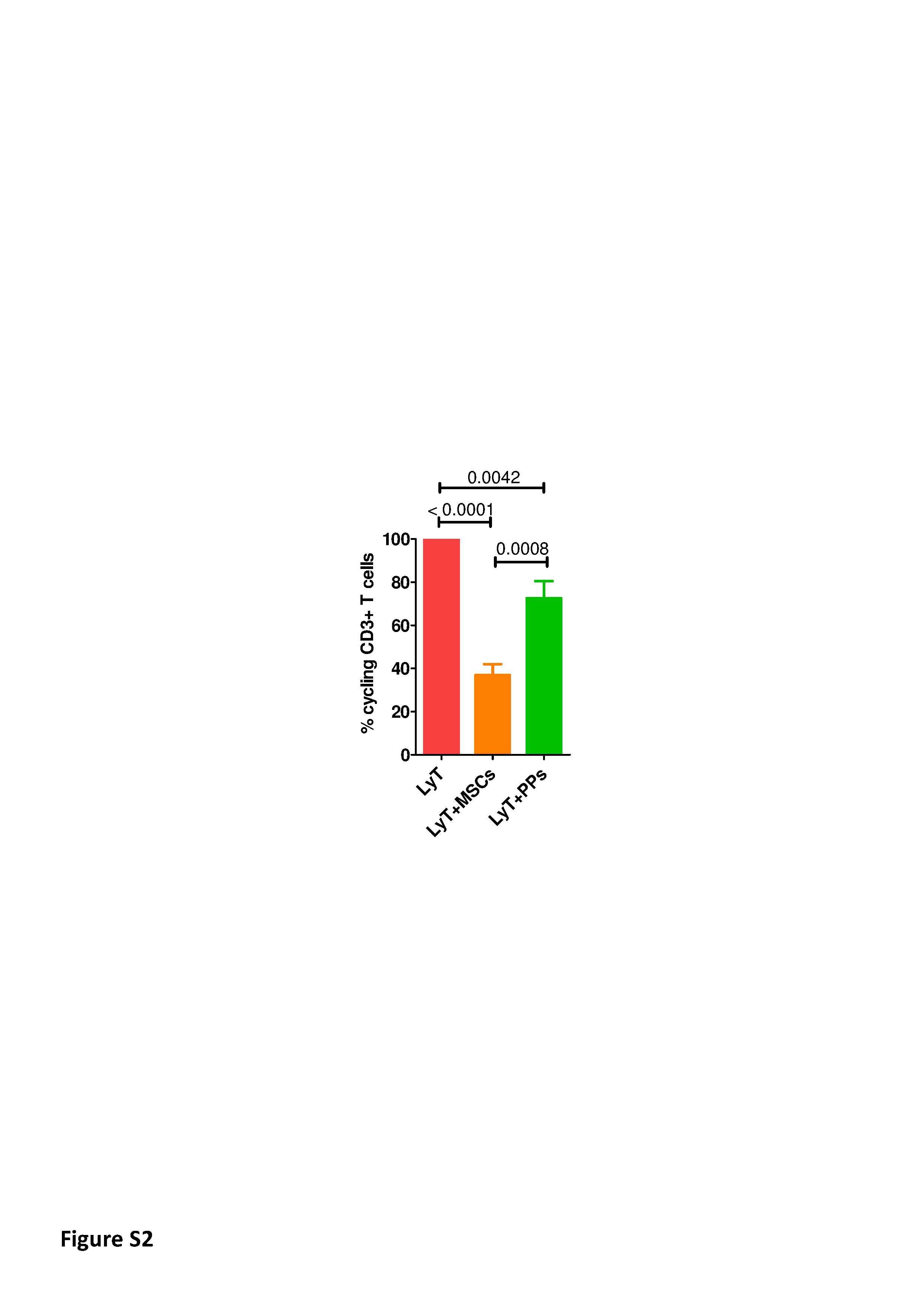

Supplement: Figure S2 — Immunosuppression potential assay of PPs and MSCs. For in vitro immunosuppression assay 50×103 cells cultured in EGM-2 or MSM were co-cultured with 100×103 CD3+ T cells isolated from PBMCs by use of a T-cell purification kit (Miltenyi Biotec, Bergish Gladbach, Germany). We used 3 donors for MSCs or PPs and 3 donors for PBMCs. Before the co-cultures, CD3+ cells were labelled with 5-(and-6)-carboxyfluorescein diacetate, succinimidyl ester (CFSE, Invitrogen) as fluorescent cell-tracing reagent. T cells were then activated with CD3+CD28+ microbeads (Miltenyi Biotec). After 5 days, all cells were recovered, and T cells were stained with anti-CD3 and anti-CD45 antibodies (Miltenyi Biotec). The proportion of cycling CD3+CD45+ T cells was quantified by fluorescence decrease of CFSE as compared with both uncycling cells (non-stimulated cells) and stimulated T cells cultured without MSCs or PPs. Analysis involved use of the Cyan™ flow cytometer (Beckman Coulter, Villepinte, France) and Kaluza™ software. The percentage of CD3+ T lymphocytes proliferating during the co-cultures (LyT + MSCs or LyT + PPs) were calculated. Stimulated T lymphocytes without MSCs or PPs (LyT) were used as control cells, for 100% of cycling cells. Data are mean ± SEM from 3 experiments. (TIF) [file pone.0048648.s002.tif]
